# An examination of the socio-demographic correlates of patient adherence to self-management behaviors and the mediating roles of health attitudes and self-efficacy among patients with coexisting type 2 diabetes and hypertension

**DOI:** 10.1186/s12889-020-09274-4

**Published:** 2020-08-12

**Authors:** Zhenzhen Xie, Kaifeng Liu, Calvin Or, Jiayin Chen, Mian Yan, Hailiang Wang

**Affiliations:** 1 Department of Industrial and Manufacturing Systems Engineering, The University of Hong Kong, Hong Kong, China; 2 School of Intelligent Systems Science and Engineering, Jinan University, Zhuhai, China; 3 Department of Systems Engineering and Engineering Management, City University of Hong Kong, Hong Kong, China

**Keywords:** Self-management, Type 2 diabetes mellitus, Hypertension, Self-efficacy, Health attitudes, Socio-demographic correlates, Health behavior

## Abstract

**Background:**

Patients with coexisting type 2 diabetes and hypertension generally exhibit poor adherence to self-management, which adversely affects their disease control. Therefore, identification of the factors related to patient adherence is warranted. In this study, we aimed to examine (i) the socio-demographic correlates of patient adherence to a set of self-management behaviors relevant to type 2 diabetes and hypertension, namely, medication therapy, diet therapy, exercise, tobacco and alcohol avoidance, stress reduction, and self-monitoring/self-care, and (ii) whether health attitudes and self-efficacy in performing self-management mediated the associations between socio-demographic characteristics and adherence.

**Methods:**

We performed a secondary analysis of data collected in a randomized controlled trial. The sample comprised 148 patients with coexisting type 2 diabetes mellitus and hypertension. Data were collected by a questionnaire and analyzed using logistic regression.

**Results:**

Female patients were found to be less likely to exercise regularly (odds ratio [OR] = 0.49, *P* = 0.03) and more likely to avoid tobacco and alcohol (OR = 9.87, *P* < 0.001) than male patients. Older patients were found to be more likely to adhere to diet therapy (OR = 2.21, *P* = 0.01) and self-monitoring/self-care (OR = 2.17, *P* = 0.02). Patients living with family or others (e.g., caregivers) were found to be more likely to exercise regularly (OR = 3.44, *P* = 0.02) and less likely to avoid tobacco and alcohol (OR = 0.10, *P* = 0.04) than those living alone. Patients with better perceived health status were found to be more likely to adhere to medication therapy (OR = 2.02, *P* = 0.03). Patients with longer diabetes duration (OR = 2.33, *P* = 0.01) were found to be more likely to adhere to self-monitoring/self-care. Self-efficacy was found to mediate the association between older age and better adherence to diet therapy, while no significant mediating effects were found for health attitudes.

**Conclusions:**

Adherence to self-management was found to be associated with socio-demographic characteristics (sex, age, living status, perceived health status, and diabetes duration). Self-efficacy was an important mediator in some of these associations, suggesting that patient adherence may be improved by increasing patients’ self-management efficacy, such as by patient empowerment, collaborative care, or enhanced patient–physician interactions.

## Background

The control of coexisting type 2 diabetes and hypertension requires diligent daily self-management, consisting of the use of prescribed medication, regular self-monitoring, a healthy diet, and performing regular exercise [1]. However, patients do not always adhere to these self-management behaviors [2–5]. As a result, undesired health consequences can occur, such as suboptimal therapeutic outcomes, higher odds of hospitalization, and increased mortality rates [6–8]. Such non-adherence problems and harmful consequences underscore the need to identify the patients that are most likely to exhibit non-adherence and to propose strategies to ameliorating this [9].

To date, studies on patient non-adherence with chronic disease self-management behaviors have largely focused on medication non-adherence, which has been found to be negatively associated with age [10–15], perceived health status [10, 11], education level [13], and income [10, 13]. A few studies have also reported findings regarding non-adherence to other self-management behaviors, such as the associations between a shorter diabetes duration and poorer adherence to dietary recommendations [16], between younger age and a reduced likelihood of glucose self-monitoring, and between female sex and lower levels of exercise performance [15]. Although these studies have indicated the patient groups that are less likely to adhere to self-management, some other relevant self-management behaviors have not been examined and the underlying reasons for non-adherence have not been specifically tested. Therefore, in this study, in addition to examining the adherence to previously unexplored self-management behaviors, we also aimed to assess whether non-adherence can be explained by health attitudes and self-efficacy in performing self-management, which are two crucial factors that can fundamentally change and maintain health behaviors [17–19].

Health attitudes refer to “the extent to which health concerns are integrated into a person’s daily activities” (p. 275) [20]. Studies have found that there are links between socio-demographic characteristics (e.g., being older, being female, and having a higher socioeconomic status and a tertiary education) and more positive health attitudes [21] and between positive attitudes and adherence to self-management [22–24]. These findings suggest that health attitudes may explain/mediate the association between socio-demographic characteristics and adherence to self-management. A study conducted among healthy people also found that attitudes toward healthy eating partially explained/mediated the association between education level and healthy eating behavior [25]. However, to our knowledge, no studies have examined the mediating role of health attitudes between socio-demographic characteristics and the adherence to self-management among type 2 diabetic and hypertensive patients.

Self-efficacy, defined as one’s belief in the ability to perform a behavior required to produce a desired outcome [26], is a crucial factor that may explain adherence to health behaviors in general [18, 19, 27], or explain patient adherence to self-management in particular [28]. Studies of patients with type 2 diabetes have reported associations between lower self-efficacy and poorer adherence to medication, diet, exercise, blood glucose self-monitoring, and foot care [12, 29, 30]. It has also been found that self-efficacy varies across different socio-demographic groups [18]. These findings suggest that self-efficacy in performing self-management may explain the observed differences in patient adherence across these socio-demographic groups. A study that examined the role of self-efficacy in shaping self-management behaviors among diabetic patients reported that self-efficacy partially explained the association of diabetes knowledge with adherence and fully explained the association of distress and education level with adherence [31].

Despite the evidence linking socio-demographic characteristics to health attitudes and self-efficacy, and the evidence linking health attitudes and self-efficacy to patient adherence, very few studies have examined whether health attitudes and self-efficacy mediate the associations between socio-demographic characteristics and patient adherence. In this study, we aimed to examine (i) the associations between socio-demographic characteristics (sex, age, education level, living status, perceived health status, diabetes duration, and hypertension duration) and adherence to each of the following six self-management behaviors among patients with type 2 diabetes and hypertension: medication therapy, diet therapy, regular exercise, tobacco and alcohol avoidance, stress reduction, and self-monitoring/self-care, and (ii) to examine whether health attitudes and self-efficacy explained/mediated these associations.

## Methods

### Data source

This study was a secondary data analysis of a 24-week randomized controlled trial (RCT) that evaluated the effectiveness of a tablet-based self-monitoring system in improving patient health-related outcomes, in comparison with usual care (ClinicalTrials.gov registry number: NCT02799953) [32]. The RCT protocol was pilot-tested for feasibility and quality assurance [33]. Study participants were type 2 diabetic and hypertensive patients recruited from two diabetes clinics (one clinic from each of two public hospitals). In the recruitment process, patients attending the clinics were invited to join a briefing session, during which trained research assistants (RAs) introduced the study to the attending patients, identified their eligibility, and solicited their participation. Patients were included if they were aged 18 years or above, had received a physician-confirmed diagnosis of type 2 diabetes and hypertension at least one month previously, were receiving prescribed medications for these two chronic conditions, and were able to self-manage/monitor these conditions. Baseline data of the participants in the usual care group (*n* = 148) were analyzed to explore the aims of this study. Data collection and study follow-up took place in the participants’ homes. Study variables of interest were collected using a questionnaire. One RA read each questionnaire item aloud to the patient and provided them with a printed response scale, and another RA collected the patient’s response.

### Measurements

All of the study variables were measured using items and scales adapted from previous studies. They were translated from English to Chinese using a six-step modified back-translation approach [34] and were pilot-tested for clarity and comprehensibility. Appendix Table A1 presents the items that were used to collect the socio-demographic characteristics (sex, age, education level, living status, diabetes duration, hypertension duration, and perceived health status). Perceived health status was determined by whether patients agreed that they were in good health, rated on a 7-point response scale from 1 (very strongly disagree) to 7 (very strongly agree). We assessed health attitudes using a scale with five items measured on a 7-point response scale from 1 (very strongly disagree) to 7 (very strongly agree), where each item presented a statement about positive health attitudes (e.g., “you do everything you can to stay healthy”) with which patients indicated whether they agreed [35]. The internal consistency of this scale, as measured by Cronbach’s α, was 0.72 in a previous relevant study [35] and 0.74 in this study. Self-efficacy in performing self-management was measured using a 5-item scale (e.g., “how confident are you in your ability to follow a low-salt and low-fat diet?”), with responses ranging from 1 (not at all confident) to 10 (totally confident) [36]. The Cronbach’s α of this scale was 0.89 in a previous relevant study [37] and 0.78 in this study. The Medical Outcomes Study disease-specific adherence scale [1] was used to examine adherence to medication therapy (one item), diet therapy (three items) (Cronbach’s α = 0.76), regular exercise (one item), tobacco and alcohol avoidance (two items) (Cronbach’s α = 0.32), stress reduction (one item), and self-monitoring/self-care (four items) (Cronbach’s α = 0.51). Participants responded to the items (e.g., “how often have you followed a low-salt diet in the past 2 months?”) on a 6-point scale ranging from 1 (none of the time) to 6 (all of the time) in terms of how often they performed the self-management behaviors.

### Data analysis

Descriptive statistics were calculated for the sample characteristics. Median cut-offs were used to dichotomize continuous variables (age, diabetes duration, and hypertension duration) and ordinal variables (education level [lower vs. higher], perceived health status [poor vs. good], health attitudes [less positive vs. more positive], and self-efficacy [lower vs. higher]) to obtain the most equal group sizes. Living status (a categorical variable) was stratified as living with family/others vs. living alone, because patients who live with family/others, in contrast to those who live alone, may be more socially engaged, less likely to be depressed, and receive more instrumental and emotional support, all of which may affect their health behaviors [38, 39].

To test the socio-demographic correlates of adherence to each self-management behavior (i.e., the first study aim), we performed ordered logistic regression with socio-demographic characteristics as the independent variables and adherence to the self-management behavior as the dependent variable. To confirm that the mediator (health attitudes or self-efficacy) explained/mediated the associations between socio-demographic characteristics and adherence to the behavior (i.e., the second study aim), three conditions had to be met: (i) the socio-demographic characteristics were significantly associated with the mediator; (ii) the mediator was significantly associated with adherence; and (iii) the association between socio-demographic characteristics and adherence that was found significant in the test of the first aim decreased or became nonsignificant. We used logistic regression to examine the first condition, and the second and third conditions were determined by ordered logistic regression with socio-demographic characteristics and the mediator as the independent variables and adherence to the behavior as the dependent variable. All of the analyses were performed using Stata version 14 (StataCorp LLC, Texas, USA).

## Results

### Sample characteristics

Table 1 presents the socio-demographic characteristics of the sample (*n* = 148). The participants had a mean age of 63.72 years (standard deviation [SD] = 9.60) and average disease durations of 16.60 years (SD = 11.25) for diabetes and 12.91 years (SD = 9.89) for hypertension.

**Table 1:** Characteristics of the study sample

Characteristics	Number (%)
**Sex**	
Male	88 (59.5%)
Female	60 (40.5%)
**Age (years)**	
< 64	70 (47.3%)
≥ 64	78 (52.7%)
**Education level**	
Lower (i.e., lower than secondary school)	62 (41.9%)
Higher (i.e., secondary school or above)	86 (58.1%)
**Living status**	
Living alone	15 (10.1%)
Living with family/others	133 (89.9%)
**Perceived health status**	
Poor (i.e., rating ≤ 3)	59 (39.9%)
Good (i.e., rating > 3)	89 (60.1%)
**Diabetes duration (years)**	
≤ 15	78 (52.7%)
> 15	70 (47.3%)
**Hypertension duration (years)**	
≤ 10	80 (54.1%)
> 10	68 (45.9%)

### Socio-demographic correlates of patient adherence

Table 2 presents the regression analysis results (i.e., odds ratios [OR], 95% confidence interval [CI], and *p*-values) for the associations between socio-demographic characteristics and patient adherence. Female patients were found to be significantly less likely to exercise regularly (OR = 0.49, *P* = 0.03) and more likely to avoid tobacco and alcohol (OR = 9.87, *P* < 0.001) than male patients. Older patients were found to be significantly more likely to exhibit better adherence to diet therapy (OR = 2.21, *P* = 0.01), and self-monitoring/self-care (OR = 2.17, *P* = 0.02). Patients living with family/others were found to be significantly more likely to exercise regularly (OR = 3.44, *P* = 0.02) and significantly less likely to avoid tobacco and alcohol than those living alone (OR = 0.10, *P* = 0.04). Patients with better perceived health status were found to be significantly more likely to adhere to medication therapy (OR = 2.02, *P* = 0.03). Patients with longer diabetes duration were found to be significantly more likely to adhere to self-monitoring/self-care (OR = 2.33, *P* = 0.01). Education level and hypertension duration were not found to be significantly associated with patient adherence.

**Table 2:** Associations between socio-demographic characteristics and patient adherence

**Socio-demographic characteristics**	**Adherence to medication therapy**		**Adherence to diet therapy**		**Adherence to regular exercise**	
	OR (95% CI)	*P*	OR (95% CI)	*P*	OR (95% CI)	*P*
**Sex**						
Male	1		1		1	
Female	1.55 (0.81, 2.96)	0.19	1.33 (0.72, 2.46)	0.36	0.49 (0.26, 0.94)	0.03*
**Age (years)**						
< 64	1		1		1	
≥ 64	1.21 (0.65, 2.28)	0.55	2.21 (1.19, 4.09)	0.01*	1.17 (0.63, 2.20)	0.62
**Education level**						
Lower	1		1		1	
Higher	0.66 (0.34, 1.29)	0.23	1.23 (0.66, 2.30)	0.51	1.84 (0.95, 3.59)	0.07
**Living status**						
Living alone	1		1		1	
Living with family/others	0.63 (0.23, 1.69)	0.36	0.74 (0.29, 1.87)	0.52	3.44 (1.24, 9.49)	0.02*
**Perceived health status**						
Poor	1		1		1	
Good	2.02 (1.08, 3.76)	0.03*	1.44 (0.80, 2.61)	0.23	1.69 (0.91, 3.15)	0.10
**Diabetes duration (years)**						
≤ 15	1		1		1	
> 15	0.99 (0.53, 1.87)	0.99	1.08 (0.59, 1.97)	0.81	1.72 (0.92, 3.20)	0.09
**Hypertension duration (years)**						
≤ 10	1		1		1	
> 10	1.58 (0.83, 3.01)	0.17	0.90 (0.49, 1.66)	0.74	0.66 (0.35, 1.24)	0.20
**Socio-demographic characteristics**	**Adherence to tobacco and alcohol avoidance**		**Adherence to stress reduction**		**Adherence to self-monitoring/self-care**	
	OR (95% CI)	*P*	OR (95% CI)	*P*	OR (95% CI)	*P*
**Sex**						
Male	1		1		1	
Female	9.87 (3.83, 25.41)	< 0.001*	0.60 (0.32, 1.12)	0.11	1.23 (0.68, 2.24)	0.49
**Age (years)**						
< 64	1		1		1	
≥ 64	1.73 (0.79, 3.78)	0.17	0.76 (0.41, 1.41)	0.39	2.17 (1.16, 4.05)	0.02*
**Education level**						
Lower	1		1		1	
Higher	1.08 (0.50, 2.34)	0.84	1.62 (0.84, 3.11)	0.15	1.00 (0.55, 1.83)	1.00
**Living status**						
Living alone	1		1		1	
Living with family/others	0.10 (0.01, 0.85)	0.04*	0.75 (0.28, 2.00)	0.57	2.37 (0.94, 5.97)	0.07
**Perceived health status**						
Poor	1		1		1	
VGood	1.87 (0.87, 3.99)	0.11	1.61 (0.87, 2.96)	0.13	0.81 (0.45, 1.46)	0.49
**Diabetes duration (years)**						
≤ 15	1		1		1	
> 15	0.88 (0.41, 1.87)	0.74	1.68 (0.90, 3.16)	0.10	2.33 (1.25, 4.37)	0.01^*^
**Hypertension duration (years)**						
≤ 10	1		1		1	
> 10	1.32 (0.60, 2.92)	0.49	1.18 (0.62, 2.24)	0.61	0.77 (0.41, 1.45)	0.42

^*^indicates a significant association

### Mediating role of health attitudes and self-efficacy

#### Results for the first condition of the mediating effect

Table 3 presents the regression results for the associations of socio-demographic characteristics with health attitudes and self-efficacy. Notably, patients with better perceived health status were found to be significantly more likely to have more positive health attitudes (OR = 2.70, *P* = 0.01). Older patients (OR = 2.61, *P* = 0.01) and patients with a higher education level (OR = 2.14, *P* = 0.05) were found to be significantly more likely to report higher self-efficacy in performing self-management.

**Table 3:** Associations of socio-demographic characteristics with health attitudes and self-efficacy

Socio-demographic characteristics	Health attitudes		Self-efficacy	
	OR (95% CI)	*P*	OR (95% CI)	*P*
**Sex**				
Male	1		1	
Female	0.97 (0.46, 2.03)	0.93	1.18 (0.57, 2.46)	0.66
**Age (years)**				
< 64	1		1	
≥ 64	2.06 (0.97, 4.37)	0.06	2.61 (1.25, 5.45)	0.01*
**Education level**				
Lower	1		1	
Higher	1.62 (0.76, 3.48)	0.21	2.14 (1, 4.56)	0.05*
**Living status**				
Living alone	1		1	
Living with family/others	0.59 (0.18, 1.89)	0.37	1.02 (0.32, 3.19)	0.98
**Perceived health status**				
Poor	1		1	
Good	2.70 (1.32, 5.52)	0.01*	1.85 (0.91, 3.75)	0.09
**Diabetes duration (years)**				
≤ 15	1		1	
> 15	2.04 (0.95, 4.37)	0.07	1.36 (0.65, 2.85)	0.42
**Hypertension duration (years)**				
≤ 10	1		1	
> 10	0.55 (0.25, 1.21)	0.14	1.14 (0.54, 2.43)	0.73

*indicates a significant association

#### Results for the second condition of the mediating effect

Table 4 presents the associations between the mediators (i.e., health attitudes and self-efficacy) and patient adherence (socio-demographic characteristics were controlled). Health attitudes were not found to be significantly associated with patient adherence. In contrast, patients with higher self-efficacy were found to be significantly more likely to exhibit better adherence to medication therapy (OR = 2.76, *P* = 0.002), diet therapy (OR = 3.57, *P* < 0.001), and regular exercise (OR = 2.30, *P* = 0.01).

**Table 4:** Associations between mediators and patient adherence (socio-demographic characteristics were controlled)

**Variables**	**Adherence to medication therapy**		**Adherence to diet therapy**	
	OR (95% CI)	*P*	OR (95% CI)	*P*
**Health attitudes**				
Less positive	1		1	
More positive	0.65 (0.34, 1.24)	0.19	0.88 (0.48, 1.62)	0.69
**Self-efficacy**				
Lower	1		1	
Higher	2.76 (1.45, 5.26)	0.002*	3.57 (1.91, 6.65)	< 0.001*
**Variables**	**Adherence to regular exercise**		**Adherence to tobacco and alcohol avoidance**	
	OR (95% CI)	*P*	OR (95% CI)	*P*
**Health attitudes**				
Less positive	1		1	
More positive	0.92 (0.49, 1.73)	0.79	1.20 (0.55, 2.59)	0.65
**Self-efficacy**				
Lower	1		1	
Higher	2.30 (1.22, 4.32)	0.01*	0.95 (0.44, 2.04)	0.89
**Variables**	**Adherence to stress reduction**		**Adherence to self-monitoring/self-care**	
	OR (95% CI)	*P*	OR (95% CI)	*P*
**Health attitudes**				
Less positive	1		1	
More positive	1.11 (0.58, 2.10)	0.75	0.86 (0.47, 1.59)	0.64
**Self-efficacy**				
Lower	1		1	
Higher	1.36 (0.73, 2.53)	0.34	1.28 (0.70, 2.35)	0.42

*indicates a significant association

#### Results for the third condition of the mediating effect

Table 5 presents the associations between socio-demographic characteristics and patient adherence with the mediators controlled. We observed that the significant association between older age and better adherence to diet therapy (as in Table 2, OR = 2.21, *P* = 0.01) became nonsignificant after controlling for the mediators (as in Table 5, OR = 1.72, *P* = 0.10). We also observed that the following associations decreased after controlling for the mediators: the association between age and adherence to self-monitoring/self-care (decreased from OR = 2.17, *P* = 0.02 to OR = 2.09, *P* = 0.03), the association between living status and adherence to regular exercise (decreased from OR = 3.44, *P* = 0.02 to OR = 3.19, *P* = 0.03), and the association between perceived health status and adherence to medication therapy (decreased from OR = 2.02, *P* = 0.03 to OR = 1.98, *P* = 0.04).

**Table 5:** Associations between socio-demographic characteristics and patient adherence after controlling for mediators

**Variables**	**Adherence to medication therapy**		**Adherence to diet therapy**		**Adherence to regular exercise**	
	OR (95% CI)	*P*	OR (95% CI)	*P*	OR (95% CI)	*P*
**Sex**						
Male	1		1		1	
Female	1.47 (0.77, 2.81)	0.25	1.33 (0.72, 2.45)	0.36	0.46 (0.24, 0.88)	0.02
**Age (years)**						
< 64	1		1		1	
≥ 64	1.08 (0.57, 2.05)	0.82	1.72 (0.91, 3.28)	0.10^#^	1.00 (0.52, 1.90)	0.99
**Education level**						
Lower	1		1		1	
Higher	0.57 (0.29, 1.12)	0.11	1.07 (0.58, 1.99)	0.83	1.66 (0.85, 3.26)	0.14
**Living status**						
Living alone	1		1		1	
Living with family/others	0.59 (0.22, 1.59)	0.30	0.72 (0.29, 1.79)	0.48	3.19 (1.14, 8.91)	0.03^#^
**Perceived health status**						
Poor	1		1		1	
Good	1.98 (1.03, 3.80)	0.04^#^	1.29 (0.70, 2.38)	0.41	1.62 (0.85, 3.08)	0.14
**Diabetes duration (years)**						
≤ 15	1		1		1	
> 15	0.96 (0.50, 1.84)	0.90	1.02 (0.55, 1.90)	0.95	1.68 (0.89, 3.16)	0.11
**Hypertension duration (years)**						
≤ 10	1		1		1	
> 10	1.50 (0.78, 2.88)	0.22	0.85 (0.45, 1.61)	0.63	0.63 (0.33, 1.19)	0.15
**Variables**	**Adherence to tobacco and alcohol avoidance**		**Adherence to stress reduction**		**Adherence to self-monitoring/self-care**	
	OR (95% CI)	*P*	OR (95% CI)	*P*	OR (95% CI)	*P*
**Sex**						
Male	1		1		1	
Female	10.03 (3.88, 25.91)	< 0.001	0.58 (0.31, 1.10)	0.10	1.22 (0.67, 2.21)	0.51
**Age (years)**						
< 64	1		1		1	
≥ 64	1.71 (0.76, 3.83)	0.20	0.69 (0.36, 1.32)	0.26	2.09 (1.09, 4.02)	0.03^#^
**Education level**						
Lower	1		1		1	
Higher	1.08 (0.49, 2.35)	0.85	1.54 (0.79, 2.98)	0.21	0.97 (0.53, 1.78)	0.92
**Living status**						
Living alone	1		1		1	
Living with family/others	0.10 (0.01, 0.84)	0.03	0.76 (0.28, 2.02)	0.58	2.30 (0.91, 5.84)	0.08
**Perceived health status**						
Poor	1		1		1	
Good	1.80 (0.83, 3.94)	0.14	1.52 (0.81, 2.85)	0.19	0.81 (0.44, 1.48)	0.50
**Diabetes duration (years)**						
≤ 15	1		1		1	
> 15	0.87 (0.40, 1.86)	0.71	1.62 (0.86, 3.07)	0.14	2.36 (1.25, 4.46)	0.01
**Hypertension duration (years)**						
≤ 10	1		1		1	
> 10	1.36 (0.61, 3.03)	0.46	1.19 (0.62, 2.28)	0.60	0.77 (0.41, 1.45)	0.42

^#^ indicates that the significant associations between socio-demographic characteristics and patient adherence (as in Table 2) decreased or became nonsignificant after controlling for the mediators (as in Table 5)

#### Summary of the mediating effects

An examination of all three conditions showed that health attitudes did not mediate any association between socio-demographic characteristics and patient adherence, but self-efficacy did mediate the association between older age and better adherence to diet therapy.

## Discussion

### Main findings

This study showed that there were significant associations between some socio-demographic characteristics (sex, age, living status, perceived health status, and diabetes duration) and adherence to several diabetes and hypertension self-management behaviors (medication therapy, diet therapy, regular exercise, tobacco and alcohol avoidance, and self-monitoring/self-care). It also showed that self-efficacy in performing self-management significantly mediated the association between age and adherence to diet therapy.

Notably, we found that female patients were less likely to exercise regularly than male patients, consistent with previous observations [40–42]. Possibly, women shoulder more responsibilities for taking care of the family, which leaves them with less time for exercise [41–43]. Women also reported more barriers to exercise (e.g., a lack of safe and appropriate sports facilities, fear of injury during exercise, and a lack of relevant skills and knowledge) than their male counterparts [40, 41]. Interventions that may help overcome these barriers, such as the provision of safe and easily accessible sports facilities, and the provision of adequate training for women to learn to exercise safely, should be considered. Another reason for the comparatively low exercise level of women may be that women received less social support (e.g., from family and friends) for performing physical activities, which is known to be an important determinant of exercise adherence [44]. In addition, lack of an exercise companion is also a commonly cited reason for not being physically active, particularly among women [45]. To help improve adherence to exercise, it has been suggested that interventions (e.g., technology and group-based exercise programs) be designed to enable the connection of companions/caregivers with patients, such that patients are encouraged and motivated to exercise [46–48].

We also found that male patients were less likely to reduce their alcohol consumption and smoking than female patients. This may be because social interactions between men are more likely to involve tobacco and alcohol [49]. Also, men may perceive smoking and alcohol consumption to be desirable masculine behaviors [50]. Another explanation may be that men are more likely to use tobacco and alcohol as a (maladaptive) coping mechanism to deal with stress [51]. If so, this may be ameliorated by interventions that promote the use of adaptive coping strategies (e.g., exercise, and talking to family and friends) to actively cope with stress [52]. Research has also shown that the perceived risk of negative consequences resulting from smoking and drinking alcohol is much smaller among men [53], suggesting that male patients need to be further educated about and alerted to the consequences of smoking and drinking alcohol.

The observed associations of younger age with poorer adherence to diet therapy and self-monitoring/self-care and the finding that the former correlation was mediated by self-efficacy in performing self-management may be attributable to the time and effort required to perform self-management. A previous study reported that “not having enough time” may be a major hindrance to the performance of self-management [54]. Therefore, older patients, who may have fewer work responsibilities and thus more spare time, may be more willing to or may find it less challenging to perform disease-related self-management [55]. Another study suggested that the daily lives of older people are more ordered and predictable [56, 57]. Accordingly, older patients may find it easier to integrate regular self-monitoring or other self-care behaviors into their more routine daily lives.

We further observed that patients who lived alone were less likely to perform physical exercise than those who lived with family/others. This may have resulted from the latter group receiving more social support from their family or companions [58]. A previous study reported that family members can provide patients with reminders and assistance to support self-management behaviors, as well as emotional support [59]. All of these factors may enhance adherence to self-management behaviors. However, it was found that patients who lived with family/others were less likely to avoid smoking and alcohol than those who lived alone. This may be due to the possible smoking or drinking behaviors of cohabitating family members, which would make it more difficult for the patients to avoid such behaviors [60]. This is consistent with previous findings that individuals’ health behaviors are strongly influenced by the lifestyles of their close associates (e.g., family members and friends) [61]. Our findings and those of previous studies suggest that the self-management behaviors of patients are strongly influenced by the people with whom they live.

We also found that patients who reported a longer diabetes duration were more likely to adhere to self-monitoring/self-care practices, possibly because they had more regularly attended clinics for follow-up consultations. This is consistent with previous reports of a positive association between the number of follow-up consultations with a physician and patient adherence [14, 62]. We infer that more follow-up visits with a physician may enable patients to gain more knowledge about their disease and health condition and thus increase their motivation to perform self-management.

We further observed an association between better perceived health status and better adherence to medication therapy, which is consistent with previous studies [11, 63–65]. This may be attributable to patients with better perceived health status having better levels of physical functioning [66] and thus being more capable of performing self-management [67]. Another possible explanation is that patients with better perceived health status may have more positive health attitudes, leading to their exhibiting enhanced adherence to medication therapy [11]. This explanation is partly supported by our finding that better perceived health status was related to more positive health attitudes, but we did not find a significant association between health attitudes and patient adherence. Further validation is needed of the interrelationships among perceived health status, health attitudes, and patient adherence to self-management.

Our analysis showed that higher self-efficacy in performing self-management was related to better adherence to diet therapy and medication therapy and greater participation in regular exercise. Patients who expressed a higher level of self-efficacy perceived themselves to be more capable of self-management and thus made greater efforts to perform self-management. In contrast, patients who expressed a lower level of self-efficacy perceived themselves to be less capable of self-management, which presumably meant that they were more likely to cease performing self-management at an early stage [68]. Our findings are therefore consistent with those of previous studies and further emphasize the role of self-efficacy as a major determinant of adherence to a healthy diet and physical exercise [12, 69–71]. We infer that improving patients’ self-efficacy in performing self-management may improve patients’ adherence to the above-described self-management behaviors [27].

In addition, we found that self-efficacy mediated the association between older age and better adherence to diet therapy, suggesting that a lack of self-efficacy was a reason for poor adherence to healthy diets among younger patients. Therefore, for this patient group, interventions that increase their self-efficacy in maintaining a healthy diet, such as by helping them make informed decisions about diet plans, enhancing their capability to cope with interruptions of diet plans, and eliciting appropriate social support for maintaining a healthy diet, may be effective in improving their adherence to diet therapy [72]. In a further analysis of the mediating effect of self-efficacy, we found that a higher education level was related to greater self-efficacy, but this did not translate to improved adherence to self-management. This finding is different from that of a previous study where self-efficacy mediated the association between education level and self-management behaviors [31]. Further research into the role of self-efficacy in shaping patient adherence to self-management behaviors is warranted.

### Implications for future research

Type 2 diabetes and hypertension often coexist in patient populations, and there is a considerable overlap between their complications and mechanisms [73]. However, few studies have examined patients with coexisting type 2 diabetes and hypertension and even fewer have investigated these patients’ self-management strategies. Future research is warranted to investigate the barriers to and facilitators of self-management among these patients.

The self-management of type 2 diabetes and of hypertension have a number of commonalities, as both require long-term medication therapy, the maintenance of a healthy diet, regular exercise, cessation of smoking and alcohol consumption, stress reduction, and self-monitoring [1], but there may be differences in the ease with which these conditions can be managed. Future research to investigate and compare patient adherence to diabetes-specific and hypertension-specific self-management behaviors is also warranted.

The patient groups exhibiting non-adherence to different self-management behaviors varied greatly, suggesting that the factors shaping these self-management behaviors (medication therapy, diet therapy, exercise, tobacco and alcohol avoidance, and self-monitoring/self-care) may also have varied greatly. It is therefore suggested that future studies conduct in-depth investigations of the underlying reasons for patient non-adherence to each specific self-management behavior. Moreover, further research is warranted to identify the mediators between socio-demographic characteristics and patient adherence, as these would reveal the underlying reasons for non-adherence in certain socio-demographic groups and thus enable the development of bespoke strategies to effectively improve patient self-management in these groups.

As noted above, “not having enough time” may be a major hindrance to the performance of self-management, especially for patients with high levels of work and/or family responsibility and a lack of spare time. This finding suggests the need for more research to identify self-management behaviors that would yield significant health benefits while simultaneously saving time. Few studies have systematically investigated the amount of time needed to perform type 2 diabetes and hypertension self-management behaviors. Therefore, we recommend additional research in this area. We also suggest that future evaluations of self-management interventions should consider and investigate the time-consuming or time-saving nature of the interventions [54].

### Implications for practice

Given the observed associations between self-efficacy and patient adherence, we suggest that interventions intended to improve patient adherence should consider both behavioral and psychological aspects. Previous research has suggested that patient education interventions that merely provide health knowledge may not sufficiently induce the desired behavioral changes [74]. Accordingly, the incorporation of psychological components into traditional patient-education interventions may yield improvements in patient adherence. Approaches that comprise collaborative care, enhanced patient–physician interaction, and patient empowerment may improve patients’ sense of self-efficacy regarding self-management and thus improve patient adherence [75, 76]. Moreover, we suggest that these approaches should be applied to younger patients and patients with lower education levels, given the observed associations of these characteristics with poorer self-efficacy.

We examined why patients with a lower education level tended to have lower self-efficacy in performing self-management, and found in the literature that patients with lower education levels tended to have poor health literacy [77], which is significantly related to poor self-efficacy [78]. Accordingly, improving the health literacy of patients with lower education levels may be an effective approach to enhance these patients’ self-efficacy in self-management.

Although several self-management interventions have been shown to yield improvements in patient adherence, few have been specifically developed to target patients in certain socio-demographic groups or to improve adherence to certain self-management behaviors. We suggest that the development of bespoke interventions for specific patient groups would more effectively improve patient adherence. In general, the use of networking and mobile technology could be considered for these purposes [32, 33, 79–81]. More specifically, smartphone-based schedule planning and reminder systems could be developed to enable younger patients to fit self-management behaviors (e.g., self-monitoring and exercise) into their busy work schedules, and to remind and motivate them to adhere to health-care behaviors. For female patients, group-based exercise programs may be an effective intervention to promote their participation in physical exercise [47].

As a lack of social support may explain why patients who lived alone reported poorer adherence to self-management behaviors, interventions that promote social support (e.g., through community care services and patient-support groups) may effectively improve the level of adherence in this population. Analogously, the health behaviors of patients who lived with family/others may have been strongly influenced by the behaviors of their cohabitants, suggesting that interventions that educate a patient’s cohabitants about a patient’s disease, and that encourage their cohabitants to be supportive of a patient’s self-management behaviors, may improve a patient’s adherence to self-management [82, 83].

In the development and implementation of interventions that aim to promote self-management adherence, such as caregiver-assisted programs or technology-based support, the design, usability, and acceptance of the interventions should be carefully studied and considered. This is necessary to prevent avoid unintended, undesirable effects on the implementation effort or on patient outcomes due to mismatches between the interventions and patients’ needs and characteristics [33, 48, 79, 84–89].

### Limitations

This study has some limitations. The study’s exploratory nature made it difficult to form prespecified hypotheses. As it is stated in the literature that exploratory analyses without prespecified hypotheses should be conducted without multiple test adjustments, the data of this study were analyzed without multiplicity adjustment [90]. We suggest that the findings of our study be further tested and confirmed in confirmatory studies with pre-implementation power analysis, prespecified hypotheses, and multiple test adjustments.

## Conclusions

Patient adherence to self-management was found to be associated with several socio-demographic characteristics, namely, sex, age, living status, perceived health status, and diabetes duration. Based on these findings, we suggest potentially effective, bespoke interventions to improve patient adherence in the socio-demographic groups that exhibited low adherence levels in this study. Self-efficacy was found to mediate the correlation between older age and better adherence to diet therapy, and was related to patient adherence to medication therapy and regular exercise. Therefore, we suggest incorporating behavioral and psychological components, such as collaborative care, enhanced patient–physician interactions, patient empowerment, and social support, into traditional patient-education interventions, as these components may promote self-efficacy and consequently improve patient adherence. The usability and acceptance of interventions must be carefully considered to avoid negative effects from poorly designed programs.

## Data Availability

The datasets used and/or analyzed in this study are available from the corresponding author on reasonable request.

## APPENDIX

**Table A1:** Socio-demographic questionnaire

**Sex**	□ Male □ Female	
**Age**	____ years	
**Education**	□ No schooling completed □ Some primary schooling □ Completed primary school □ Some secondary schooling □ Completed secondary school □ Diploma, advanced diploma, associate degree or equivalent □ Bachelor’s degree □ Master’s degree □ Doctoral degree □ Other (please specify): _____________	
**Living status**	□ Live alone □ Live with family □ Other (please specify): _____________	
**Perceived health status**	1 – Very strongly disagree 2 – Strongly disagree 3 – Disagree 4 – Neutral 5 – Agree 6 – Strongly agree 7 – Very strongly agree	
You feel very healthy and great about yourself.	1 2 3 4 5 6 7	
Your health is excellent.	1 2 3 4 5 6 7	
**Types and duration of diagnosed chronic illnesses**	□ Diabetes	Duration since diagnosis: ____ years ____ months
	□ Hypertension	Duration since diagnosis: ____ years ____ months

## Abbreviations

CI: Confidence interval
OR: Odds ratio
RA: Research assistant
RCT: Randomized controlled trial
SD: Standard deviation
